# HIV testing and care in Burkina Faso, Kenya, Malawi and Uganda: ethics on the ground

**DOI:** 10.1186/1472-698X-13-6

**Published:** 2013-01-23

**Authors:** Carla Makhlouf Obermeyer, Sarah Bott, Ron Bayer, Alice Desclaux, Rachel Baggaley

**Affiliations:** 1 Center for Research on Population and Health, Faculty of Health Sciences, American University of Beirut, Beirut, Lebanon; 2 Independent consultant, Los Angeles, CA, USA; 3 Center for the History and Ethics of Public Health, Mailman School of Public Health, Columbia University, New York, USA; 4 Institut de Recherche pour le Développement, Dakar, Sénégal; 5 HIV/AIDS Department, World Health Organization, Geneva, Switzerland

**Keywords:** Ethics, Medical, HIV Infections/diagnosis/drug therapy/prevention & control/transmission, Informed consent, Confidentiality, Counseling, HIV Seropositivity/diagnosis/transmission, Health Services Accessibility, Adult, Health policy, Mass screening

## Abstract

**Background:**

The ethical discourse about HIV testing has undergone a profound transformation in recent years. The greater availability of antiretroviral therapy (ART) has led to a global scaling up of HIV testing and counseling as a gateway to prevention, treatment and care. In response, critics raised important ethical questions, including: How do different testing policies and practices undermine or strengthen informed consent and medical confidentiality? How well do different modalities of testing provide benefits that outweigh risks of harm? To what degree do current testing policies and programs provide equitable access to HIV services? And finally, what lessons have been learned from the field about how to improve the delivery of HIV services to achieve public health objectives and protections for human rights? This article reviews the empirical evidence that has emerged to answer these questions, from four sub-Saharan African countries, namely: Burkina Faso, Kenya, Malawi and Uganda.

**Discussion:**

Expanding access to treatment and prevention in these four countries has made the biomedical benefits of HIV testing increasingly clear. But serious challenges remain with regard to protecting human rights, informed consent and ensuring linkages to care. Policy makers and practitioners are grappling with difficult ethical issues, including how to protect confidentiality, how to strengthen linkages to care, and how to provide equitable access to services, especially for most at risk populations, including men who have sex with men.

**Summary:**

The most salient policy questions about HIV testing in these countries no longer address *whether* to scale up routine PITC (and other strategies), but *how.* Instead, individuals, health care providers and policy makers are struggling with a host of difficult ethical questions about how to protect rights, maximize benefits, and mitigate risks in the face of resource scarcity.

## Background

The ethical discourse about HIV testing has undergone a profound transformation in recent years. For the decade and a half after HIV tests became available in 1985, ethical concerns centered on the right not to be tested, since an HIV diagnosis provided few medical benefits and posed serious risks of stigma and discrimination [1]. Discussions among ethicists, human rights advocates and policy makers focused on the 3-Cs: counseling, voluntary informed consent and confidentiality [2]. However, the greater availability of antiretroviral therapy (ART), and growing evidence that ART can prevent transmission of HIV have strengthened public health arguments for scaling up access to testing as a gateway to prevention, treatment and care [3,4]. Thus, the policy discourse shifted away from the right not to be tested to the right to know. In 2006, United Nations (UN) Member States committed themselves to universal access to treatment, which required a dramatic scale up in testing [5]. By 2007, WHO (World Health Organization) and UNAIDS (The Joint United Nations Programme on HIV/AIDS) guidelines recommended that all patients in settings with generalized epidemics be offered HIV testing routinely during clinical encounters – also known as provider-initiated counseling and testing (PITC) – and proposed streamlined counseling and consent procedures [2]. By 2010, 32 of 38 countries with generalized HIV epidemics had national guidelines advising health care providers to initiate testing and counseling in all clinical encounters [6]. Countries have invested in a range of other testing strategies as well, including stand-alone sites, mobile, home-based, index, and self-testing, as well as one-off and annual campaigns [6].

This expansion of testing prompted debates within the bioethics and human rights literature [7]. Critics asked whether routine PITC would threaten fundamental rights to voluntary, informed consent and confidentiality, whether confidentiality would be protected in overstretched health care facilities, whether clinical settings would provide adequate post-test counseling, support and linkages to treatment, and whether benefits would outweigh the risks of adverse consequences such as stigma, rejection and spousal abuse [8-15]. There have also been questions about how to balance HIV-positive individuals' right to medical confidentiality with the need to prevent transmission to others and to diagnose partners living with HIV [16,17]. In addition, there have been concerns about how to ensure equitable access to testing and treatment for those who face barriers to testing and care in sub-Saharan Africa, particularly most at risk groups [18,19].

Based on the limited but growing body of empirical evidence that has begun to shed light on these questions, this paper reviews what is known from the public health, human rights and policy literature about the ethical dimensions of expanding HIV testing in sub-Saharan Africa. To illustrate the evolution of testing policies and their implementation on the ground, we focus on four sub-Saharan African countries, namely: Burkina Faso, Kenya, Malawi and Uganda, selected to complement field research of the MATCH (Multi-country African Testing and Counseling for HIV) study, which investigated clients' and providers' experiences of HIV testing across different testing modalities in these countries.

## Methods

The literature searches for this review were conducted among a wide range of sources within the public health, social science literature, ethics and human rights literature. Search engines and databases such as PubMed, POPLINE and Google Scholar were scanned for the following key terms: HIV testing and counseling, PITC, routine testing, HIV disclosure, confidentiality, consent, partner notification, mandatory testing, HIV policies and legislation, criminalization of transmission, human rights, as well for sources specific to each of the four MATCH study countries. We included any paper that presented data about testing programs and policies in the 4 countries or that discussed different approaches to testing and the implementation of testing programs. Many sources had been identified in the course of preparing earlier papers on HIV disclosure, consent, and the human rights implications of different modes of delivering HIV testing and counseling. In addition, this review drew on documents, reports and guidelines published by United Nations (UN) agencies such as the Joint United Nations Programme on HIV/AIDS (UNAIDS), the World Health Organization (WHO), and non-governmental organizations (NGOs).

By reviewing evidence from four countries, we direct attention to sources that provided insight into “Prime;ethics on the ground” rather than to more theoretical discussions that have been the focus of much of the international literature. To this end, this paper is structured in three parts: a) a description of how testing policies and practices have changed in the four countries; b) a review of evidence about what ethical implications these changes have for the health, rights and wellbeing of individuals, families and communities; and finally c) a synthesis of key ethical challenges and research gaps that should be addressed. This paper focuses on those issues most relevant for adults living with HIV, as some issues particular to children or adolescents are beyond the scope of this review.

## Review

### Expansion of HIV testing and counseling in Burkina Faso, Kenya, Malawi, and Uganda

Burkina Faso, Kenya, Malawi, and Uganda exemplify different epidemiological profiles, cultural contexts, policies and health service responses [6]. All four have generalized epidemics, but as noted in Table 1, the estimated HIV prevalence among adults aged 15-49 ranges from 1.2% in Burkina Faso, to 6.3% in Kenya, 6.5% in Uganda, and 11% in Malawi [20].

**Table 1:** Selected HIV indicators for Burkina Faso, Kenya, Malawi and Uganda, 2009 and 2010

	**Estimated prevalence of HIV among adults aged 15-49, 2009**		**Estimated percentage of adults aged 15+ who received HIV testing and counseling in the past 12 months, 2010**	**Estimated percentage of pregnant women tested for HIV, 2010**	**Estimated ART coverage (among all age groups) based on 2010 WHO guidelines, 2010**	
**Country**	**%**	**[Range]**	**%**	**%**	**%**	**[Range]**
Burkina Faso	1.2	[1.0-1.5]	7.3	54	49	[44-55]
Kenya	6.3	[5.8-6.5]	29.1	83	61	[56-66]
Malawi	11.0	[10.0-12.1]	25.8	66	na	[49-57]
Uganda	6.5	[5.9-6.9]	18.1	63	47	[43-51]

Source: [6,20].

Before HIV treatment became more widely available, most people who tested in these countries (and globally) did so in the context of client-initiated 'voluntary testing and counseling' (VCT). Utilization of testing was low, both as a proportion of those estimated to be living with HIV and relative to the proportion who said they would like to know their status, as documented in Kenya, Malawi and Uganda [18,21-23]. Certain population groups faced particular barriers to testing. For example, women were less likely than men to use VCT in parts of Kenya and Uganda [24], and less likely than men to receive services in the course of testing campaigns in Burkina Faso [25]. Barriers to testing for both women and men included fear of stigma and lack of confidentiality, long distances to VCT sites, lack of perceived benefits, and delays in receiving results (before rapid testing) [21].

As HIV treatment became more widely available, all four countries expanded access to testing, each following its own unique trajectory. Three of the four countries received PEPFAR funding for HIV/AIDS prevention, treatment and care. Between 2004 and 2009, Kenya and Uganda received the largest amounts, approximately USD 1.9 million and USD 1.2 million respectively, while Malawi received about USD 0.16 million [26]. Malawi used decentralization and task shifting to lower level health personnel to scale up testing in the context of resource constraints [27,28]. Burkina Faso expanded HIV services through a unique network of community-based organizations, in partnership with government and international organizations [29,30]; they also carried out large national testing campaigns [31]. Kenya set ambitious annual targets and launched large door-to-door home-based testing programs, outreach and mobile services for most-at risk populations, as well as integrated rural campaigns that combined HIV testing, malaria and diarrhea prevention [32-34]. Uganda also used a diversified portfolio of approaches, including campaigns, VCT, PITC and large home-based testing efforts in rural areas [35,36].

**Figure 1:**
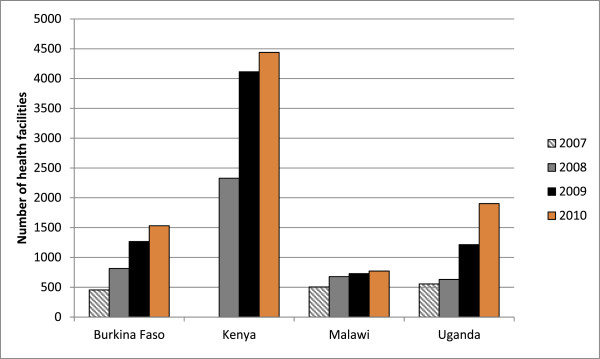
**Estimated number of health facilities providing HIV testing and counseling, by country and year.** Notes: 1. No estimate is available for Kenya 2007. 2. Source for 2007-2009 data: [40]; source for 2010 data: [6] .

Much expansion of testing in these countries occurred through increased PITC within clinical services such as antenatal care (ANC), tuberculosis care, and hospital inpatient settings. In fact, national guidelines recommending PITC in clinical settings were issued in Kenya [37], Malawi [38], and Uganda [39], even before WHO and UNAIDS published their 2007 guidelines [2]. In Burkina Faso, PITC was expanded more recently (around 2007), though it was available earlier at some health facilities. As illustrated by Figure 1, statistics show remarkable increases in the number of testing sites in all four countries in recent years [6,40]. In Kenya, for example, the number of testing sites increased from 3 in 2000 to more than 4,000 in 2009 [6,41]. As a result, by 2009, the proportion of health facilities offering routine PITC had reached an estimated 73% of facilities [41]. Similarly, in Uganda, they increased from 3 in 2002 to more than 1,200 by 2009 [5].

**Figure 2:**
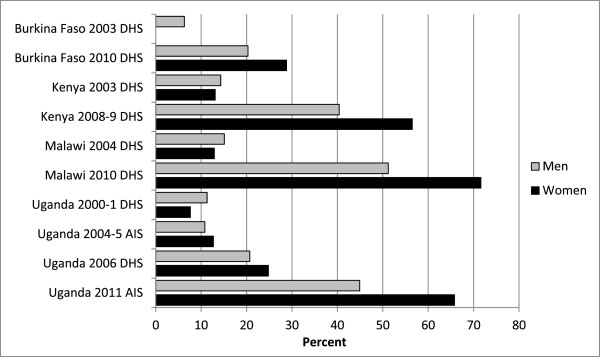
**Percentage of adults aged 15-49 who ever had an HIV test and received results, DHS and AIS surveys 2000-2011.** Sources: Demographic (DHS) and AIDS Indicator Survey (AIS) final reports [43-50]. Note however that the Burkina Faso 2003 DHS and Uganda 2000-1 DHS final reports did not include these indicators in a comparable format, so those figures were drawn from the comparative analysis of DHS data by Mishra and colleagues [42] .

As a result of this expansion, the number and percentage of individuals ever tested has also increased over the past decade, as illustrated by findings from population-based surveys in all four countries, as presented in Figure 2[42-50]. As noted in Table 1, by 2010, the WHO estimates that the percentage of individuals aged 15 and older who had received an HIV test and results in the past 12 months had risen to 7% in Burkina Faso, 29% in Kenya, 26% in Malawi, and 18% in Uganda [6,51].

### Ethical dimensions of expanding testing and counseling

#### Does routine PITC undermine voluntary informed consent?

One important question about expanding routine PITC was whether it would undermine voluntary informed consent. Critics suggested that clients, especially women in antenatal care, might perceive routine PITC as mandatory; that power differentials between patients and providers would make it difficult for patients to refuse testing; and that streamlined pre-test procedures might weaken the quality of counseling [12].

While PITC was designed to increase testing in all health facilities, including those providing inpatient services, tuberculosis care, and male circumcision, most available evidence on consent comes from studies of antenatal care facilities. Predictions that routine testing in clinical settings would increase utilization of testing have been borne out. When the Mbale Regional Hospital in Eastern Uganda shifted from a client-initiated, opt in approach to a routine PITC approach in 2006, rates of testing increased from 20% to 87.6% of attendees [52]. Similarly dramatic increases in testing uptake within ANC were documented in Burkina Faso [30], Kenya [53], Malawi [27] and other settings in Uganda [52,54]. By 2010, the estimated proportion of pregnant women tested nationally had risen to 54% in Burkina Faso, 66% in Malawi, 83% in Kenya and 63% in Uganda [6]; and in some facilities with routine PITC, acceptance rates exceeded 95% [27,53,55].

Earlier studies cited high uptake of testing through PITC as evidence of “acceptability” [56,57], though it was acknowledged that returning for results (required before rapid tests were available) was much lower, suggesting a degree of ambivalence about testing. More recently, while some researchers, including from Malawi, concluded that they found “no evidence of coercion” [27], high acceptance rates raise the possibility that routine PITC is difficult for pregnant women to refuse. Indeed, several studies from Burkina Faso, Kenya, Malawi, and Uganda have found that while many women report feeling no pressure to test, many others believe that HIV testing in ANC is compulsory, feel they cannot refuse the advice of a physician, and are not clearly told that testing is optional; they feel pressured by staff who heavily emphasize their responsibility and the benefits for their baby [53,58-61].

A number of studies have concluded that the quality and comprehensiveness of counseling varies widely: patients in antenatal care and other services are sometimes given inadequate information about their right to decline, the consent process and the benefits and rationale for HIV testing, and they are not always given time to ask questions or make decisions, as documented in Burkina Faso [62], Kenya [63] and the MATCH study countries [64].

It may however be problematic to assume that women who previously did not seek out or who declined testing before routine PITC were exercising informed, autonomous choice. Decisions about testing are complex, and studies have documented many reasons why pregnant women do not test or receive results (whether in client-initiated VCT or when offered a routine test by a provider), including low risk awareness, fear of stigma, lack of perceived benefits, time and cost burdens. Before testing within ANC became routine, an important barrier to testing in all four countries was that many women felt they could not test without their husband's or family's permission, whom they often believed would oppose testing; this was reported in Burkina Faso [62], Kenya [24], Malawi [22], and Uganda [65-69]. Similarly, in Malawi, men told researchers that HIV testing by a wife without her husband's consent was a valid reason for divorce [70]. Evidence suggests that the introduction of routine testing within antenatal care can make it easier for women who want to test to do so with less fear of partner or family opposition, as reported in studies from Burkina Faso [71], Kenya [53,68,69], Malawi [70] and Uganda [65,72]. In explaining the dramatic increase in acceptance of HIV testing following the introduction of routine PITC in Lilongwe, Malawi, researchers wrote: “the new ANC attendees were less fearful of accepting HIV testing because routine testing was perceived by their partners and families as standard of care given to all pregnant women in the clinic” [52]. Similarly, a study in Uganda found that routine testing made it easier for women to test without their husband's permission, because testing was seen as being done for the baby's health [72].

This evidence suggests two possible changes: a) routine testing policies may encourage women to test without asking for partner or family permission; and b) the policy shift may increase partners' and families' support for HIV testing. Routinizing testing may thus contribute to shifting the decision from the moral realm—testing because of reprehensible behavior—to the health realm—testing as a standard part of prenatal care and for the baby's health. It is likely that similar shifts have occurred in other health facilities, and that where testing is provider-initiated, it loses some of its exceptional nature and becomes a routine medical procedure.

#### Does PITC lead to benefits? Access to prevention, support and treatment

The ethical and public health rationale for routine testing depends heavily on the potential for diagnosis to lead to prevention and treatment [4]. Thus, one key question is whether those who test positive are able to access counseling, support, treatment and care following diagnosis. Many have voiced concern that PITC policies may be accompanied by reduced levels of counseling and high levels of loss to follow up, both of which weaken the rationale for scaled up testing [11,12,73].

More than a decade ago, research had indicated that HIV testing and counseling could contribute to reducing risk behaviors among those who tested both negative and positive [74]. One concern has been that lower levels of counseling in PITC programs would undermine this preventive potential. In fact, some research, including a study from three districts in Kenya, Tanzania, and Zambia found that counseling provided in the context of PITC was “limited” and concluded that the services missed important opportunities for prevention [63]. This is a particular concern both for those who are HIV-negative, and for those who test positive for HIV, but do not qualify for treatment under current guidelines.

**Figure 3:**
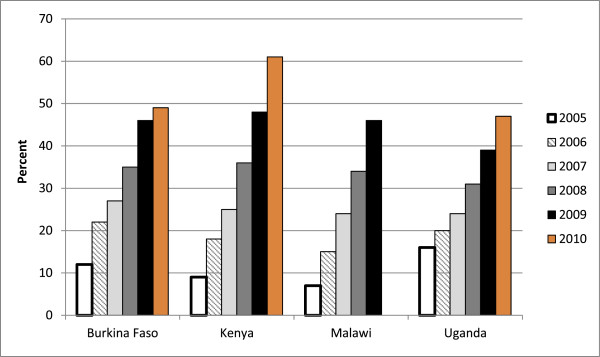
**WHO estimates of ART coverage among all age groups 2005-2010, by country and year.** Notes: This indicator is defined as the percentage of adults and children with advanced HIV infection currently receiving ART in accordance with nationally approved treatment protocols (or WHO/UNAIDS standards) among the estimated number of adults and children with advanced HIV infection. No point estimate is available for Malawi in 2010. The estimated range is 49-57%, as noted in Table 1. Source for 2007-2009 data: [40]; source for 2010 data: [6] .

Another key question is how well these programs ensure linkages to care. In recent years, governments in all four countries have placed a high priority on expanding access to treatment [41,51,75,76]. As a result, the numbers and percentages of eligible individuals receiving treatment have increased accordingly, as illustrated by Figure 3[6,40]. Nonetheless, barriers to treatment pose continuing challenges in all four countries. A systematic review of evidence from sub-Saharan African countries (including Kenya, Malawi and Uganda) found substantial loss to follow up at every stage between HIV diagnosis, pre-ART care, and ART initiation [77], as have studies from Burkina Faso [78,79], Kenya [80-83], Malawi [19,84-86], and Uganda [87-89].

Measuring loss to follow-up can be challenging, given high mobility of patients in the four countries. In addition, rates of attrition after HIV diagnosis are often difficult to compare across studies, due to different definitions of linkages to care (e.g. whether provision of cotrimoxazole or registration at a clinic is sufficient, or whether ART is a necessary element in the package). Moreover, the situation in the field may be changing rapidly, and studies from a few years ago may not reflect current conditions. Typical, however, is a study from Kenya among women diagnosed with HIV in a PMTCT (Prevention of mother to child transmission) program, which found that after exiting the program, half had changed residence; 74% of those located reported going to the HIV referral program, but 33% subsequently discontinued care, mostly because they did not qualify for treatment [82]. Another study among adults already eligible for ART in Kenya and Malawi found high levels of attrition both before and after starting ART, either through loss to follow-up, death, or stopping treatment, including 23% of patients in Malawi and 15% in Kenya during the pre-ART phase, and another 26% in Malawi and 23% in Kenya after beginning ART; overall program attrition was 43% [80].

A host of factors contribute to attrition after diagnosis, including policies that render many recently diagnosed HIV-positive patients ineligible for treatment, the challenge of following patients who do not yet qualify for ART, problems with pre-ART referral systems [77,90], and generally weak health systems. In some settings, ART stock-outs, inadequate pre-antiretroviral care and a lack of staff confidentiality also appear to pose barriers to care [91]. In a study from Kenya, women who did not access care after being diagnosed in PMTCT cited concerns about confidentiality and poor quality services [82]. In addition, attending pre-ART care before treatment eligibility may involve high costs for HIV-positive individuals, both in terms of money and time, often with few perceived benefits [77]. In Burkina Faso, pre- ART patients who wish to become eligible for treatment often must pay for laboratory monitoring, transport and accommodation, which present barriers to care [92]. In Kenya, cost of transport was an important reason why treatment eligible respondents said they had not started ART [93]. In Uganda, 39% of patients in one study had dropped out of treatment after enrollment by year three, and while some had sought treatment elsewhere, those who did not cited lack of transportation and work/child care responsibilities as primary reasons [94].

Social and cultural factors also play a role in loss to follow-up. In Malawi, HIV-positive women cited a broad range of reasons for dropping out of PMTCT or pre-ART services, including fears of negative reactions from partners, friends and community, unequal gender relations, and lack of support from husbands, as well as fatalism about HIV [84,95,96]. In another study from Malawi, HIV-positive men reported postponing treatment because of masculine ideals of strength, because they feared losing the respect of friends or partners, or because of concerns that entering treatment would reduce their chances of (re)marriage [96]. In some cases, women in Kenya dropped out of PMTCT because they believed their children had been healed through faith or traditional medicine [97]. Another study from Kenya found that women who had not accessed care were less likely to believe that ART was effective or to have disclosed their HIV status to their partners [82]. In fact, most studies found that fear of stigma and lack of disclosure to family and friends were important barriers to HIV care following diagnosis. Lack of disclosure also poses a barrier to preventive behaviors, such as breastfeeding with early weaning or formula feeding, as documented in studies from Burkina Faso [98] and Malawi [85].

Health programs from the region have implemented many strategies to improve linkages to care and retention in pre-ART services and to better define what should be provided to those HIV-positive but not eligible for ART. Some provide point of care CD4 testing and prophylaxis for opportunistic infections [77]; others, such AMPATH in western Kenya, have found that loss to follow up has declined over time, possibly because they have increased the number of parent and satellite clinics, provided more comprehensive care (e.g. giving food supplements) and enhanced community outreach efforts [99].

Governments have also tried various policies and strategies to improve access to care. Burkina Faso recently eliminated user fees for ART [92]. Malawi introduced simplified initiation criteria for pregnant women [90], decentralized HIV service delivery and shifted responsibility for ART initiations to non-physician clinicians—a strategy that was linked to a doubling of ART enrollment in some districts [28]. These efforts are promising, and increased numbers of individuals receiving treatment represents progress. On the other hand, some have expressed concern that reduced international funding for HIV treatment programs globally may undermine these efforts, through increased treatment stock-outs, restrictions in enrolment of new patients, and the re-emergence of ART user-fees in settings such as Burkina Faso [92].

#### Does PITC lead to adverse consequences?

A major ethical concern in debates about expanded testing is whether adverse consequences that sometimes follow an HIV diagnosis will outweigh benefits, especially in light of barriers to care cited above. A large body of evidence from sub-Saharan Africa indicates that fears of negative reactions by partners, families and communities pose important barriers to HIV testing, prevention and treatment, particularly for women [100]. An unintended consequence of testing during antenatal care is that women are often first to know their HIV status and hence, they may be accused of having brought HIV into a couple, as documented in an analysis of qualitative data from the MATCH study [101]. A recent study in antenatal clinics in Kenya found that while fewer than 4% of women declined the routine offer of HIV testing, substantial proportions of all 1,525 women interviewed believed they would experience serious adverse consequences if they tested positive including 28.3% who thought they would be rejected by their family, 25.6% who believed they would be physically abused by their partner, and 32.1% who thought their relationship would end [102].

While fears of negative consequences have been repeatedly documented, the extent of actual adverse events following HIV diagnosis has been more difficult to assess. A 2004 review of studies from developing countries (including two studies from Burkina Faso and one from Kenya) found that negative reactions to disclosure, such as blame, abandonment and violence, were relatively infrequent, ranging from 3-15% [103]. These low rates must be considered in light of several other factors, however. First, women selectively disclose their HIV-positive status, and they may not disclose to partners whom they expect would respond badly [17,104]. Second, HIV-positive women in serodiscordant relationships appear to be at increased risk [105]. Third, in some settings, adverse events can be more frequent. In one community in Malawi, abandonment following HIV disclosure by pregnant women was so high that the community called the PMTCT service “the divorce program” [106]. On the other hand, researchers note that because most studies are retrospective, it is not always possible to determine a causal link between the adverse events and HIV disclosure, as domestic abuse may have preceded disclosure [107]. Moreover, in Malawi, researchers suggested that divorce was not always the least desirable outcome, particularly for women who were in a bad relationship, and it could sometimes facilitate women's access to treatment, when natal families provided better care during their illness than unsupportive husbands [96]. In addition, living with undiagnosed HIV (the alternative to testing) carries its own risks, including eventual illness and possibly death, and may simply postpone negative reactions.

#### How to balance disclosure, confidentiality, and partner notification

Lack of disclosure to partners – both by women and men – poses serious ethical challenges, particularly given that considerable proportions of those living with HIV are members of serodiscordant couples, including large majorities in Burkina Faso, Kenya, and Malawi [104,108] and nearly half of those diagnosed with HIV in Uganda [109]. A recent review [17] found that disclosure rates in the region varied widely, with lower rates typically reported for partner disclosure compared to disclosure to other family members. While women and men may fear negative consequences of disclosure, secrecy carries its own burdens, including isolation, lack of support in the face of a potentially life threatening disease, and concerns about transmitting the infection to partners or children [98]. Health care providers in these countries often struggle to balance a duty to notify partners at risk with a competing ethical obligation to protect the medical confidentiality, safety and wellbeing of those living with HIV [9]. In one study in Kenya, maternity care providers found it inconceivable that a husband would not be informed if his wife tested positive for HIV [110]. When confronted with guidelines that appeared to bar breaches of confidentiality, counselors in Malawi sometimes bent the rules if they believed that family members or partners should know a client's HIV status [111,112]. In Uganda, researchers found that HIV counselors “struggled to decide how to act when members of discordant couples refused to reveal their HIV status, leaving partners or children at risk”, and they described wanting more guidance in this area [61].

Policy makers are also grappling with the dilemma of confidentiality and its limits. In Burkina Faso, a 2008 law mandates disclosure to sex partners and criminalizes transmission under certain circumstances [113]. In Uganda, proposed legislation would mirror provisions of Burkina Faso's law. There is little systematic evidence about the extent to which such laws are enforced, but some argue that women may be more vulnerable to prosecution for non-disclosure or criminal transmission than men in sub-Saharan Africa because they are more likely both to be HIV-positive and to know their status due to routine testing in ANC [114]. There is consensus within the international public health and human rights community that criminalizing nondisclosure undermines human rights and serves no useful public health benefit [115,116]. There is less consensus about the ethics of partner notification, however. In the face of nondisclosure to sexual partners, some argue that partner notification, even without patients' consent, may be “the lesser of the two evils” [16]. Guidelines issued by the UN High Commissioner for Human Rights and UNAIDS, and policies in Kenya suggest that health workers may disclose patients' HIV status without their consent if a risk of HIV transmission exists, as long as they meet certain conditions, including concealing the identity of the patient (if possible) and providing follow-up support [117,118]. In this region, however, such conditions are difficult to fulfill and little is known about how to protect HIV-positive clients who might be placed at risk through involuntary third party disclosure [119].

Partner notification and index patient testing programs have been assessed through a recent randomized trial in Malawi, and researchers concluded that it was “feasible, acceptable, and effective”, as well as virtually harmless [120]. However, some important ethical questions are left unanswered; for example, most index patients were married to their only partner, and it isn't clear to what extent the identity of the index patient could be concealed [121]. It is also noteworthy that at least some patients who were eligible for the study declined to participate because they did not want their HIV status disclosed.

### Concerns about equity

Another theme in the international ethical discourse about HIV testing is the concept of equity. Are those who need HIV services able to access them? And conversely, do some groups—particularly those at higher risk—face disproportionate barriers to testing, treatment and support [18]? Concerns about equitable access to testing have focused on a number of population groups, including men, adolescents, rural populations who lack access to health services, prisoners in custodial settings, and most-at-risk populations such as men who have sex with men, sex workers, and injecting drug users.

#### Ensuring male access to HIV testing

One striking consequence of the scale-up of testing within ANC is that in many sub-Saharan African settings, a higher proportion of women than men know their HIV-positive status and are receiving treatment; for example, according to WHO statistics, an estimated 55% of women in sub-Saharan Africa who needed antiretroviral therapy were receiving it, compared with 41% of men [6]. Gender imbalances in access to testing or treatment have also been documented in individual studies from Burkina Faso [122,123], Kenya [99], Malawi [19], and Uganda. Part of the numerical gender imbalance reflects the fact that women comprise a higher proportion of those living with HIV than men; however, in many settings, men have lower utilization of health services than women, and therefore, fewer opportunities to test and receive HIV care early in the infection, and they often delay testing and/or treatment until they become symptomatic [18,96,124]. From an equity perspective, there is also concern that testing pregnant women in ANC without testing their male partners represents a missed opportunity to diagnose or prevent transmission of HIV in light of persistent rates of nondisclosure.

Many HIV programs have sought to reach men by trying to increase testing among partners of antenatal care patients and by promoting couples counseling and other “family-centered” approaches. Programs in Malawi, Rwanda and Zambia found that community mobilization and outreach strategies increased the use of couples counseling in ANC clinics [125-127], and some programs in the four countries have reported success with a “facilitated disclosure approach” [128]. However, PMTCT programs in Africa still find it difficult to reach male partners of women in ANC care [129], as noted in studies from Burkina Faso [62], Kenya [130-132], Malawi [106], and Uganda [59,133,134]. Couple counseling has also posed challenges in terms of how couples are defined and how health programs can maximize disclosure and support, while minimizing the risk of adverse consequences, particularly for women at risk of intimate partner violence.

#### Concerns about equity: increasing access to HIV services for most-at-risk populations

Despite official commitments from all four countries to overcome disparities in testing, most-at-risk population groups, including sex workers, injecting drug users, and prisoners, but particularly men who have sex with men, continue to face barriers to access. Regional reviews note that HIV policies and programming in sub-Saharan Africa historically focused almost exclusively on heterosexual transmission, with a corresponding neglect of research, surveillance, prevention, treatment and care for men who have sex with men [135-139]. Smith and colleagues argue that this neglect stems from a context of extreme political, cultural and religious hostility towards such men in the region, where male-to-male sex is illegal in 31 countries (including in Kenya, Malawi and Uganda) and punishable by the death penalty in four countries, a provision that is not yet law in any of the MATCH countries, but is being considered in Uganda [135,140]. This context of hostility has serious negative consequences for access to HIV services. For example, in Malawi the 2010 UNGASS report suggested that men who had sex with men had less access to testing services than female sex workers and that stigma and barriers to care are rising due to recent arrests of men who have sex with men [75]. Some efforts to reach men who have sex with men are underway in Kenya, though these are still on a limited scale and have yet to be replicated in many parts of the region [136].

### What are the ethical implications of other strategies for improving access?

#### The role of campaigns

One commonly used approach to reaching underserved populations in sub-Saharan Africa has been national or local testing and counseling campaigns, including mass media awareness and mobilization, as well as campaigns that provide services directly, for example, through mobile clinics and home-based testing. Campaigns have been implemented in all four countries [141,142], but they have been particularly important in Burkina Faso where, a considerable proportion of all testing occurs during annual campaigns [31]. In Kenya, an integrated, week-long campaign reached up to 50,000 people, with high rates of testing uptake [33,143], and a media campaign to promote HIV testing and counseling between 2002 and 2005 was linked to a substantial increase in testing uptake [144]. In Malawi a week-long campaign aimed at mobilizing people to test has been carried out annually for several years; “Testing Week” has been linked to improvements in testing uptake and is currently being formally evaluated [75,145].

Campaigns have strengths and weaknesses. While it is clear that they increase awareness of potential benefits of testing, there has been little empirical investigation into whether campaigns may pressure those who—for whatever reason—do not wish to be tested. Some express concerns that those who test during campaigns are not always able to access follow-up care and prevention, as noted in Kenya [32]. On the other hand, evidence suggests that campaigns can reach underserved groups. For example, a campaign targeting sex workers in Kenya tested over 6,000 women and clients during a five-day “moonlight” campaign [41]. And in Burkina Faso, campaigns have been used to target hard to reach groups such as sex workers, men who have sex with men, and young people aged 14-25 [31]. Home-based testing campaigns were designed to address equity by overcoming barriers to attending designated clinics such as distance, cost of transport and concerns about confidentiality [146]. While a 2010 Cochrane Collaborative Report concluded that “the impact of home-based VCT on the uptake of HIV testing is unclear in developing countries” [147], considerable enthusiasm for this approach is evident. It is clear from available reports that home-based testing can overcome certain socio-economic barriers to access, particularly for those living in rural areas far from a health facility [22,32,146]. A study from rural Kenya found that a strategy of community meetings and scheduled household contacts reached nearly two-thirds of eligible adults aged 15-49 in the community, almost all of whom agreed to be tested [148]. In Kenya and Uganda, home-based strategies reached populations with low rates of prior testing and with higher CD4 counts than those tested in other settings [35,36,149-151]. Some have voiced concerns, however, that when testing within the household context, individuals may be pressured by family members to agree to testing or to disclose their results; there are also serious concerns about how to link those who test positive to follow-up care, especially in rural areas with limited access to health facilities [32].

#### Other testing and counseling strategies

In addition to campaigns and home testing, there have been a host of less well documented strategies for expanding testing in the region, including workplace testing initiatives [152], self-testing [153], and index patient testing initiatives similar to those described earlier by Brown and colleagues [120]. Each of these strategies has its own implications for consent, confidentiality and linkages to care, and the evidence base on ethical and human rights implications is still emerging.

## Discussion

This review highlights a number of findings about scaling up HIV testing in Burkina Faso, Kenya, Malawi, and Uganda. First, the scale, design, availability, and quality of HIV services are changing rapidly in these settings. As a result empirical evidence about HIV services may become outdated within a short period of time, which is one important limitation of this review. Second, there is great variety in the ways in which HIV testing and counseling services are delivered – both among and within the countries profiled in this paper. There is also a diversity of quality of care, outcomes for patients, provider practices, and the extent to which patients access treatment and care after being diagnosed with HIV. In other words, there is no monolithic “resource poor setting”. A systematic analysis of how these differences affect key ethical questions is beyond the scope of this paper, but it is an important issue for researchers to address in the future.

Regarding the ethical dimensions of HIV testing, this review suggests a number of findings. First, ethical concerns voiced at the international level do not always correspond to debates on the ground, as illustrated by the decision by governments in Kenya, Malawi, and Uganda to implement routine PITC even before WHO and UNAIDS issued their 2007 guidelines [2], despite international controversy over this approach. The most salient policy questions about HIV testing in these countries is no longer *whether* to scale up routine PITC (and other strategies), but *how.* Instead, individuals, health care providers and policy makers are struggling with a host of difficult ethical questions about how to protect rights, maximize benefits, and mitigate risks in the face of resource scarcity.

The international ethical discourse about HIV testing has focused on four main questions: how do different testing policies and practices undermine or strengthen informed consent and medical confidentiality? How well do different modalities of testing provide benefits that outweigh risks of harm? To what degree do current testing policies and programs provide equitable access to HIV services? And finally, what lessons have been learned from the field about how to improve the delivery of HIV services to achieve public health objectives and protections for human rights?

Concerns that routine PITC might undermine informed consent have some merit, as illustrated by women in ANC who believe that HIV testing is mandatory for pregnant women. On the other hand, routine PITC may have strengthened women's ability to make autonomous decisions about their own health in settings where, prior to routine PITC, women believed that they did not have the right to test without their husband's or family's consent. A comparative analysis of consent in the four MATCH countries has shown high levels of consent across modes of testing (86%), with the level only slightly lower (83%) among PMTCT testers [64]. Routine PITC has complex implications for autonomous consent: while these policies make it harder to say no for those women who wish to decline or are ambivalent, they make it easier for those who wish to know their status to say yes. This has been most clearly demonstrated for women in antenatal care, but it may apply to other groups and testing modalities as well. For example, authors of a ten country study in southern Africa concluded that large proportions of the population want to know their status but “may not feel empowered to get themselves tested” [18].

With regard to risks and benefits, some evidence suggests that negative life events following HIV disclosure are less common than once feared [107], and it is possible that stigma and discrimination may decline as access to testing and treatment expands. Meanwhile, expanding access to testing and treatment in these four countries has made the biomedical benefits of HIV testing increasingly clear for individuals, their children, and their partners. WHO estimates suggest that the greater availability of ART has helped reduce AIDS related mortality globally, and within sub-Saharan Africa specifically, where an estimated 30% fewer people died from AIDS-related causes in 2010 than in 2004, following the dramatic expansion in access to ART in that region [6]. Moreover, evidence about the potential public health benefits of testing and treatment is changing rapidly. Until recently, researchers believed that expanding testing could reduce HIV prevalence primarily by encouraging people to reduce high risk sexual behavior [4]. However, research published in 2011 found that early treatment of those who tested positive reduced transmission to partners by 96%, suggesting that testing combined with early treatment has enormous untapped potential for prevention [154]. As the knowledge base on treatment as prevention grows, policy makers will have to address questions about the ethical and public health rationale for eligibility requirements that delay treatment until patients meet criteria based on CD4 counts [155]. At the same time, individuals who test positive for HIV in these four countries often face serious barriers to care, and expanded testing will not provide full benefits for those living with HIV until health systems can address the systemic, social and economic challenges that undermine linkages to care [77].

## Conclusion

In sum, evidence from these countries suggests that the expansion of testing and counseling services—including routine PITC—can increase access to services and achieve positive health outcomes. It also suggests a myriad of ways in which HIV services need to address protections for human rights. Policy makers and practitioners are still grappling with important ethical issues. One is how to ensure equitable access to HIV testing and treatment for rural populations, adolescents, men, and high risk groups such as men who have sex with men. Another is how to balance patients' right to confidentiality with the duty to prevent transmission to partners and children at risk. The difficult ethical issues related to involuntary disclosure and partner notification have yet to be resolved in many of these settings. A third issue is what health service approaches can reduce the risk of negative consequences of disclosure, e.g. through facilitated disclosure, couples counseling, integrating attention to violence against women, and broader efforts to reduce HIV stigma. Finally, the legality, ethics, advisability and practicality of third party disclosure continue to fuel policy debates in these countries—without clear or easy solutions.

## Abbreviations

AIS: AIDS indicator survey; ANC: Antenatal care; ART: Antiretroviral therapy; DHS: Demographic and health survey; MATCH study: Multi-country African testing and counseling for HIV; PITC: Provider initiated testing and counseling; PMCTC: Prevention of mother to child transmission; UN: United Nations; UNAIDS: The Joint United Nations Programme on HIV/AIDS; VCT: Voluntary counseling and testing; WHO: World Health Organization.

## Competing interests

The authors declare no competing interests.

## Authors’ contributions

CO is the Principal Investigator on the MATCH study. She conceived of this article, oversaw the work of all authors, and participated in all aspects of reviewing the literature, writing and editing the manuscript. SB carried out the literature review, and took a lead role in writing and editing. RBayer contributed to the initial discussions, and participated in the writing and editing. AD, RBaggaley, and members of the MATCH study group (AH, OK, RW, IN, and PC) contributed to the literature review and the editing. All authors read and approved the final manuscript.

## Authors’ information

In addition to the authors of this article, MATCH (Multi-country African Testing and Counseling for HIV) Study group members, include:

Peter Cherutich, National AIDS/STD Control Program, Ministry of Health, Nairobi, Kenya. pcheru2000@yahoo.com.

Anita Hardon, Amsterdam Institute for Social Science Research, University of Amsterdam, Amsterdam, the Netherlands. ahardon@xs4all.nl

Odette Ky-zerbo, Programme d^′^Appui au Monde Associatif et Communautaire de Lutte Contre le VIH/SIDA, Ouagadougou, Burkina Faso. kyzerbo_odette@yahoo.fr.

Ireen Namakhoma, Research for Equity and Community Health Trust, Lilongwe, Malawi. inamakhoma@yahoo.co.uk.

Rhoda Wanyenze, Makerere University School of Public Health, Kampala, Uganda. rwanyenze@hotmail.com.

## Pre-publication history

The pre-publication history for this paper can be accessed here:

http://www.biomedcentral.com/1472-698X/13/6/prepub
